# Baculovirus Utilizes Cholesterol Transporter NIEMANN–Pick C1 for Host Cell Entry

**DOI:** 10.3389/fmicb.2019.02825

**Published:** 2019-12-05

**Authors:** Zhihong Li, Youpeng Fan, Junhong Wei, Xionge Mei, Qiang He, Yonghua Zhang, Tian Li, Mengxian Long, Jie Chen, Jialing Bao, Guoqing Pan, Chunfeng Li, Zeyang Zhou

**Affiliations:** ^1^State Key Laboratory of Silkworm Genome Biology, Southwest University, Chongqing, China; ^2^Department of Microbiology, Guizhou University of Traditional Chinese Medicine, Guiyang, China; ^3^Chongqing Key Laboratory of Microsporidia Infection and Control, Southwest University, Chongqing, China; ^4^Key Laboratory for Sericulture Functional Genomics Biotechnology of Agricultural Ministry, Southwest University, Chongqing, China; ^5^College of Life Sciences, Chongqing Normal University, Chongqing, China

**Keywords:** *Bombyx mori*, BmNPV, NPC1, cholesterol trafficking pathway, enveloped virus

## Abstract

The dual roles of baculovirus for the control of natural insect populations as an insecticide, and as a tool for foreign gene expression and delivery, have called for a comprehensive understanding of the molecular mechanisms governing viral infection. Here, we demonstrate that the *Bombyx mori* Niemann-Pick C1 (BmNPC1) is essential for baculovirus infection in insect cells. Both pretreatment of *B. mori* embryonic cells (BmE) with NPC1 antagonists (imipramine or U18666A) and down-regulation of NPC1 expression resulted in a significant reduction in baculovirus BmNPV (*B. mori* nuclear polyhedrosis virus) infectivity. Disruption of BmNPC1 could decrease viral entry (2 hpi) rather than reduce the viral binding to the BmE cells. Furthermore, our results showed that NPC1 domain C binds directly and specifically to the viral glycoprotein GP64, which is responsible for both receptor binding and fusion. Antibody blocking assay also revealed that the domain C specific polyclonal antibody inhibited BmNPV infection, indicating that NPC1 domain C most likely plays a role during viral fusion in endosomal compartments. Our results, combined with previous studies identifying an essential role of human NPC1 (hNPC1) in filovirus infection, suggest that the glycoprotein of several enveloped viruses possess a shared strategy of exploiting host NPC1 proteins during virus intracellular entry events.

## Introduction

Enveloped viruses include a number of pathogens with significant importance to animal or human health. As a typical double-stranded DNA enveloped virus, baculoviruses are known to infect invertebrates, with over 600 host species described (Blissard and Rohrmann, 1990; Wood and Granados, 1991). Due to a strong species-specific tropism for arthropods, baculovirus has been widely used as a biopesticide (Jiang and Xia, 2014). Concurrently, baculovirus can transduce a broad range of vertebrate cells, including human, bovine, fish avian, and even primitive cells such as embryonic stem cells, warranting its application as a biological tool for gene delivery (Volkman and Goldsmith, 1983; Kost et al., 2005; Chen et al., 2011). During its viral infection cycle, baculovirus produces two virion phenotypes: occlusion-derived virus (ODV) which transmits to insects by the oral route and specializes to infect midgut epithelial cells, and budding virus (BV) produced during nucleocapsid budding from the basolateral membrane of infected cells, responsible for systemic host infection (Slack and Arif, 2006). During viral infection, BV first attaches to the host cell surface, and is internalized by endocytosis to late endosomes (Volkman and Goldsmith, 1985); subsequently, the viral membrane of BV fuses with the endosomal membrane upon low pH trigger, and nucleocapsids are released into cytoplasm followed by viral replication (Kielian and Rey, 2006; Kadlec et al., 2008). The major BV (Group I alphabaculoviruses) envelope protein, glycoprotein 64 (GP64), has been shown to be essential for both virus attachment and membrane fusion (Rahman and Gopinathan, 2003). Although a number of host molecules including heparin sulfate (Duisit et al., 1999), phospholipids (Tani et al., 2001) or *Bombyx mori* receptor expression-enhancing protein (BmREEPa) (Dong et al., 2015) have been identified to be involved in BV attachment or binding, the exact identity of host receptors for baculovirus still remains elusive.

Among numerous baculoviruses, *B. mori* nuclear polyhedrosis virus (BmNPV) is one of the most frequently employed in the research, which showed close relatedness with *Autographa californica* multiple nucleopolyhedrovirus (AcMNPV). It has been reported previously that BmNPV infection was enhanced by *B. mori* promoting protein (BmPP), a family member of Niemann-Pick C2 (NPC2), in silkworm BoMo cells (Kanaya and Kobayashi, 2000). The addition of BmPP to culture media at a concentration of 1 μg/ml resulted in a 1000–10,000-fold increase of BmNPV production; however, the detailed molecular mechanism has not yet been fully elucidated. Human NPC2 protein functions as a central shuttle binding to Niemann-Pick C1 (NPC1) to export low density lipoprotein (LDL)-derived cholesterol from late endosomes and lysosomes to other cellular compartments (Infante et al., 2008). The role of BmPP in promoting BmNPV infection indicates that host proteins responsible for cholesterol transport may be involved in baculovirus infection. Interestingly, the infection of Ebola virus (EBOV) has proven to be dependent on cholesterol transporter NPC1, which serves as a virus intracellular receptor for filovirus entry (Carette et al., 2011; Côté et al., 2011; Miller et al., 2012). NPC1 is a ubiquitously expressed membrane protein which is mainly involved in intracellular cholesterol transport (Altmann et al., 2004). Structurally, NPC1 is comprised of three large luminal “loop” domains, A, C, and I (Carstea et al., 1997; Loftus et al., 1997). Domain A, also called the N-terminal domain (NTD), is involved in cholesterol binding and exporting cholesterol from lysosomes into the cytosol. Domain C directly and specifically binds to NPC2 and constitutes the scaffold to properly accommodate NPC2 for hydrophobic handoff of cholesterol to the pocket of NTD (Infante et al., 2008; Deffieu and Pfeffer, 2011; Gong et al., 2016). Furthermore, NPC1 domain C is able to bind to the cathepsin-primed form of Ebola glycoprotein (GPcl) (Gong et al., 2016; Han et al., 2016); the absence of domain C results in complete resistance to infection by EBOV, indicating that this domain is essential for EBOV entry. The third luminal domain I interacts with domain C, and may play a supporting role in cholesterol transport (Gong et al., 2016).

The recent findings of NPC1 protein as an intracellular receptor for filovirus, coupled with prior evidence supporting the involvement of NPC2 (BmPP) in BmNPV infection, prompted us to investigate the potential role of NPC1 homologs in BmNPV infection in insect cells. Here, we demonstrate that BV of BmNPV infection requires the expression of BmNPC1. BmE cells pre-treated with the NPC1 inhibitors imipramine or U18666A, which can mimic the molecular phenotype of NPC disease (Underwood et al., 1996; Jupatanakul et al., 2014; Wichit et al., 2017), results in marked inhibition of viral replication and production. Silencing, or knocking out of BmNPC1 expression also substantially impairs BmNPV proliferation *in vitro*, and partial rescue of the BmNPV-EGFP infection can be achieved by genetic complementation of BmNPC1 in BmNPC1-null BmE cells. We found that BmNPC1 deficiency could decrease viral entry, but not reduce the viral binding. Further, we provide evidence that BmNPC1 domain C is able to interact with the BmNPV-GP64, and anti-NPC1-C sera can block viral infection in a dose-dependent manner, suggesting that BmNPV might exploit the domain C of BmNPC1 to escape endosome. Together, these data show that BmNPC1 is an essential host factor for baculovirus infection in insect cells, and identifies a shared strategy of exploiting the host NPC1 protein by a group of enveloped viruses during virus intracellular entry events.

## Materials and Methods

### Cell Culture and Recombinant Virus

*Bombyx mori* cell line BmE cells were maintained at 28°C in Grace’s medium (Thermo Fisher Scientific, United States) supplemented with 10% (V/V) fetal bovine serum (FBS) (Thermo Fisher Scientific, United States) and 1%(V/V) penicillin-streptomycin (Pan et al., 2010). The recombinant BmNPV virus bearing an EGFP gene under the control of polyhedrin promoter was constructed by Bac-to-Bac Baculovirus Expression System according to the manufacturer’s protocol (Invitrogen, United States). The expression of EGFP can act as a reporter for monitoring viral gene expression and viral replication (Zhang et al., 2014). Recombinant viruses were propagated in BmE cells and viral titers were measured by 50% tissue culture infectious dose (TCID_50_) based on EGFP expression as described (Wu et al., 2010).

### Cloning, Protein Expression, and Purification of BmNPC1 and Antibody Production

The BmNPC1 transmembrane domain was predicted based on the TMHMM server^1^ and functional domains were predicted based on the website^2^. The full coding region of BmNPC1 was PCR amplified with specific primer (Supplementary Table S1) from a BmE cell cDNA library and then cloned into the pMD19-T vector for sequencing. The sequences of BmNPC1 extracellular loop A (31–264 amino acids) or I (881–1152 amino acids) were amplified with primer pairs BmNPC1-A-PE and BmNPC1-I-PE (Supplementary Table S1) and were then cloned into pET32a (+) plasmid for protein expression in *E. coli*. The recombinant BmNPC1-A, BmNPC1-I proteins with His tag were purified with a HisTrap HP 5 ml column (GE Healthcare, United States) according to the manufacturer’s instructions. The purified recombinant protein BmNPC1-A and BmNPC1-I were later used as an immunogen to immunize 7-week-old BALB/c mice with Freund’s complete adjuvant (Sigma-Aldrich, United States). The TDPVELWASPTSRS polypeptide from BmNPC1 (409–422 amino acids) coupled with the KLH tag were synthesized by GenScript and used to immunize New Zealand rabbits with Titermax Gold adjuvant. The anti-BmNPC1-A and anti-BmNPC1-I mouse serum, anti-BmNPC1 rabbit serum, and control sera from both naïve mice and rabbits immunized with KLH tag only were collected and stored at −20°C until use.

### Immunoblot Analysis

The protein samples were separated by SDS-PAGE and then transferred to polyvinylidene difluoride (PVDF) membranes (Roche, Switzerland). PVDF membranes were blocked with 5% skimmed milk diluted in TBST (150 mM NaCl, 20 mM Tris–HCl, 0.05% Tween-20) for 1 h at 37°C or at 4°C overnight. After incubation for 1 h with a 1:3000 dilution of PcAb-hNPC1 in 5% skimmed milk, each PVDF membrane was washed three times with TBST. Primary antibody binding was detected with a 1:5000 dilution of peroxidase labeled goat anti-rabbit IgG (Sigma-Aldrich, United States) in 5% skimmed milk (1 h, RT), and each PVDF membrane was washed three times. Blots were detected with ECL Plus Western Blotting Detection Reagents (Bio-Rad, United States).

### Immunofluorescence Assay

For the cell surface location analysis of BmNPC1, BmE cells were seeded on cover glasses in 12-well plates (Corning, United States) for 12 h at 28°C, and incubated with PcAb-BmNPC1-C antibody or negative rabbit IgG for 8 h at 4°C. After washing three times with TBST, the cells were fixed by 4% paraformaldehyde at room temperature for 30 min followed. Then the cells were incubated with Alexa Fluor 488-labeled anti-rabbit IgG antibody for 1 h at room temperature. After washing three times with TBST, the cells were further treated with DiI (2 μg/ml, Thermo Fisher Scientific, United States) for 20 min staining at room temperature. Unbound DiI were removed by washing three times with TBST, and the cells were imaged with an Olympus confocal microscope. For the intracellular location analysis of BmNPC1, BmE cells were transfected with vectors which could express Rab7-mCherry to label the late endosomes (Spence et al., 2016). After 48 h, transfected BmE cells were seeded on cover slips in 12-well plates (Corning, United States) for 12 h at 28°C, and fixed by 4% paraformaldehyde at room temperature for 30 min followed by permeabilization with 0.2% Triton X-100. Then the cells were incubated with PcAb-BmNPC1-C antibody or negative rabbit IgG for further analysis as described previously (Yang et al., 2017), and imaged with an Olympus confocal microscope.

### U18666A and Imipramine Treatment

BmE cells (2 × 10^5^ cells/well) were seeded into twelve-well culture plates and pre-incubated with vehicle or various concentrations of imipramine dissolved in methanol (25, 50, 75 or 100 μM) or U18666A dissolved in serum-free Grace medium (0.5, 1, 5 or 10 μM) for 2 or 24 h, respectively. Drug pre-treated cells were subjected to BmNPV-EGFP virus infection at an MOI of 1 for 72 h in the presence of drug, and viral infectivity was evaluated by EGFP positive cells, and quantification of the relative amount of viral DNA by qPCR.

### Filipin Staining

Extemporaneous preparation of the filipin staining solution described as previously (Vanier and Latour, 2015). BmE cells were seeded on cover glasses in 12-well plates (Corning, United States) for 12 h at 28°C, and treated with imipramine (100 μM for 2 h) or U18666A (10 μM for 24 h). The cells were rinsed by two washes (5 min each) with PBS. 0.5 mL/well filipin staining solution were added. Plates were left in the dark for 45 min. Then all wells were rinsed twice for 3–5 min with PBS, and the preparations were mounted in Olympus confocal microscope.

### BmNPC1-RNAi Construction and Transfection in BmE Cells

The RNAi target prediction of BmNPC1 was performed according to the website^3^; two regions designated npc1-a and npc1-b were chosen based on scores for subsequent use. The sense and antisense fragments of npc1-a and npc1-b were amplified from the BmE genome with primer pairs npc1-RNAi-a and npc1-RNAi-b (Supplementary Table S1), and were subsequently cloned into the RNAi vector pSL1180 with A3 intron as a spacer (Figure 3A), generating RNAi vectors psl-BmNPC1-a and psl-BmNPC1-b. Unmodified pSL1180 vector designated as psl-null was used as a negative control for RNAi.

BmE cells (7 × 10^5^ cells/well) grown in six-well culture plates were transfected with 2 μg of psl-BmNPC1-a or psl-BmNPC1-b, or negative control psl-null siRNA, using 2 μl of X-treme GENE HP DNA transfection reagent (Roche, Germany) following the manufacturer’s protocol for 48 h before cells were subjected to viral infection. The knockdown effect of RNAi transfection was confirmed by mRNA expression via qPCR with primer set BmNPC1-q (Supplementary Table S1).

### Construction and Verification of NPC1-Knockout (ΔNPC1) BmE Cells

The CRISPR/Cas9 genomic editing tool was used to generate the stable BmNPC1-knockout BmE cell line. The Cas9 expression vector (named pSL1180-IE1-Cas9) and sgRNA expression vector (named PUC57-U6-gRNA) were constructed as previously described (Dong et al., 2016) (Supplementary Figure S3A). The primer sequences generating the guide RNA (gRNA) targeting the BmNPC1 gene were designed based on the CRISPR website^4^; all candidate sgRNA target sequences bear the GN19NGG sequence. Next, extraction of two optimal target sites (gRNA-1 and gRNA-2) based on the CRISPR website (see text footnote 4)was performed; the forward and reverse primers of the target sequence start with AAGT and AAAC, respectively. The corresponding oligos were annealed and ligated into a sgRNA expression vector which was digested by *Bbs*I to generate sgRNA-1 and sgRNA-2 expression plasmids. All sgRNA expression plasmids were sequenced by M13 primers for verification. For construction of the BmNPC1 replacement donor vector, homologous arms targeted upstream of the gRNA-1 site and downstream of the gRNA-2 site were amplified from *B. mori* genomic DNA and designated as Left-HR and Right-HR, respectively. The selective markers ie1-DsRed-SV40 and A3-neo-SV40 were maintained in our laboratory. The Left-HR, DsRed expression cassettes, neomycin (neo) expression cassettes, and Right-HR were sequentially cloned into the pBluescript II KS (-) vector to generate the donor plasmid pKS-donor (Supplementary Figure S3B). All clones were verified by sequencing of plasmids. The primers are listed in Supplementary Table S1.

### Reverse Transcriptase PCR and Quantitative Real-Time PCR (qPCR)

Total RNA from BmE cell was extracted using Total RNA kit II (Omega, United States) following manufacturer’s protocol, and reverse transcription was carried out using MLV Reverse Transcriptase (Promega, United States). These cDNA samples were used to detect transcripts of BmNPC1 using the primers BmNPC1-q (Supplementary Table S1). The BmRPL3 (gi|112982798) amplified with primers BmRPL3-q (Supplementary Table S1) was used as the internal reference. Sample analysis was performed on the CFX96^TM^ Real-Time System (Biorad, United States).

The viral DNA loads were calculated based on qPCR of the BmNPV *gp41* gene. Total DNA from each sample was prepared with a Wizard Genomic DNA extraction Kit (Promega, United States) according to the manufacturer’s protocol, and the qPCR was performed using primers *gp41*-q (Supplementary Table S1) targeting a 120-bp region of the BmNPV *gp41* gene (AAC63752.1). The *Bmgapdg* gene amplified by primers BmGAPHG-q (Supplementary Table S1) was used as the reference.

### Viral Infection and Quantification of Infectivity

Wild type or ΔNPC1-BmE cells (7 × 10^5^ cells/well) seeded into six-well culture plates were inoculated with BmNPV-EGFP virus at an MOI of 1 for 2 h, after which cells were washed three times with serum-free Grace medium followed by incubation with Grace medium supplemented with 10% FBS. Viral infectivity was monitored by EGFP expression of positive cells at the indicated time point. The mean percentage of EGFP positive cells from five representative fields was calculated, and the data presented are representative of three independent experiments. The viral DNA loads in cells were determined by relative quantification of viral *gp41* gene using qPCR and were expressed as the fold of change compared to the corresponding control. Viral titers in RNAi-NPC1 cells were determined with a TCID_50_ endpoint dilution assay based on the EGFP expression of positive cells.

### Virus Binding and Internalization Assay

Wild type or ΔNPC1-BmE cells were seeded at 7 × 10^5^ cells/well in six-well culture plates 1 day prior to infection. The cells were pre-cooled at 4°C for 30 min, then inoculated with BmNPV-EGFP virus (MOI = 10) at 4°C in Grace medium containing 10% FBS for 1 h. For viral binding assay, cells were washed three times with PBS at 4°C to remove dissociative virus particles before total DNA extraction. For viral internalization assay, cells were washed three times with serum-free Grace medium at 4°C to remove unbound viruses and cultured at 28°C for another 2 h. Total DNA from each sample was prepared with a Wizard Genomic DNA extraction Kit (Promega, United States) according to the manufacturer’s protocol, and the viral DNA loads in cells were determined by qPCR at the indicated time points.

### *In vitro* Expression of BmNPC1 and GP64 Proteins and Co-immunoprecipitation

Fragments of BmNPC1-A, BmNPC1-C, BmNPC1-I and BmNPV GP64 were amplified and cloned into pT7CFE1-NHis-GST-CHA vector (Thermo Fisher Scientific, United States) for subsequent expression by 1-Step CHO High-Yield *in vitro* Translation (IVT) Kit following the manufacturer’s protocol (Thermo Fisher Scientific, United States). A, C, and I-domains of BmNPC1 were expressed with His_9_-tag and GST-tag to the N-terminus and a HA-tag to the C-terminus; GP64 protein were only expressed with His_9_-tag and GST-tag to the N-terminus (not include a HA-tag). The expression of recombinant proteins was confirmed by Western blot using anti-HA monoclonal antibody (Sigma-Aldrich, United States) or anti-GP64 monoclonal antibody (Abcam, United Kingdom). Co-immunoprecipitation was performed in accordance with standard protocols to probe the interaction between GP64 and BmNPC1 proteins. In brief, the protein G agarose beads (Bio-Rad, United States) bearing GP64 protein through mouse anti-GP64 antibody were incubated with NPC1-A, NPC1-C or NPC1-I protein overnight at 4°C with gentle shaking. Proteins eluted from the beads were probed with anti-HA monoclonal antibody via Western blot. Co-immunoprecipitation was also performed in a reciprocal manner, in which NPC1-A, NPC1-C, and NPC1-I conjugated beads were incubated with GP64 protein, with proteins eluted from the beads probed with mouse anti-GP64 monoclonal antibody.

### Yeast Two-Hybrid Assay

The yeast two-hybrid assay was used to confirm the interaction between NPC1-A, NPC1-C, NPC1-I, and GP64 *in vivo* according to the previously described (Chen et al., 2016). The bait and prey constructs pairs pGBKT7-GP64/pGADT7- NPC1-A, pGBKT7- GP64/pGADT7- NPC1-C, pGBKT7- GP64/pGADT7- NPC1-I, and pGBKT7- NPC1-C/pGADT7-GP64 were transformed simultaneously into competent yeast cells to examine the protein interaction. All primers used are listed in Supplementary Table S1.

### Antibody Blocking Assay

BmE cells (2 × 10^5^ cells/well) seeded in twelve-well culture plates were incubated with one of the following antibodies: mouse anti-NPC1-A, anti-NPC1-I polyclonal antibody, rabbit anti-NPC1-C polyclonal antibody, mouse or rabbit naïve serum, at a concentration of 10 μg/ml for 2 h at 28°C, then the cells were inoculated with BmNPV-EGFP at an MOI of 1 for 2 h, and washed three times with serum-free Grace medium followed by incubation with Grace medium supplemented with 10% FBS. At 72 h p. i. infected cells were imaged with fluorescence microscope, and the cells were harvested for measuring viral DNA load by qPCR as described previously.

### Statistics

Independent-samples *T*-tests or ANOVA followed by Dunn’s Multiple Comparison Test were used for statistical analysis. Significant differences are marked with ^∗^*P* < 0.05, ^∗∗^*P* < 0.01, and ^∗∗∗^*P* < 0.001; n.s, non-significant, respectively. All results are graphed as means ± SD for triplicate samples. All the data presented are representative of a minimum of three independent experiments.

### Ethics Statement

All animal experiments were conducted in accordance with Laboratory Animals Ethics Review Committee of Southwest University guidelines (Chongqing, China) with committee approval for this study (Permit Number: AERCSWU2017-7). Mice were maintained in accordance with recommendations of the committee for the purpose of control and supervision of experiments on animals.

## Results

### Identification of npc1 Homologs in the Bombyx Mori Genome

**FIGURE 1:**
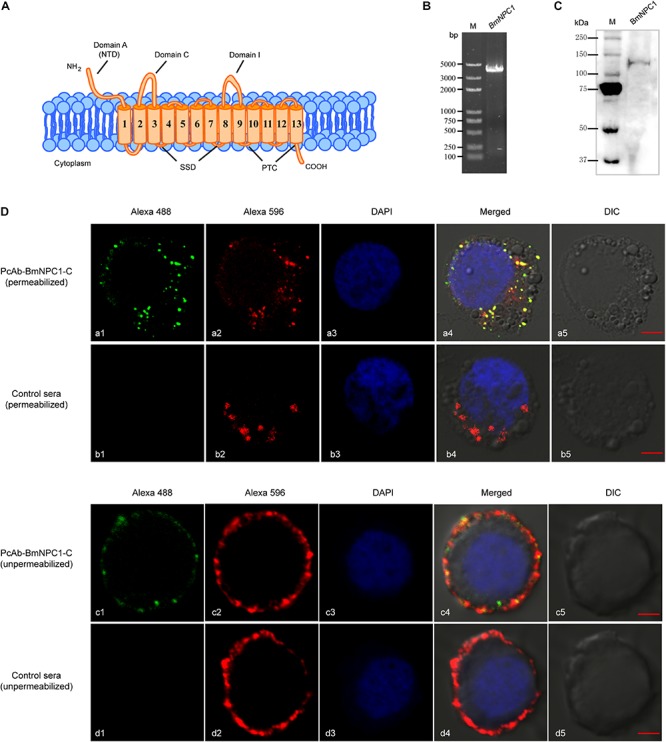
Expression and localization of BmNPC1 in BmE cell. **(A)** Schemes show the main structural characteristics of BmNPC1. BmNPC1 contained 13 transmembrane helices and 3 large conservation domains: N-terminal domain (NTD), sterol sensing domain (SSD), and patched domain (PTC). The three large luminal loops were named as domains A, C, and I. **(B)** BmNPC1 full-length gene was PCR amplified with specific primers from BmE cell cDNA. **(C)** Immunoblot of BmNPC1 in testis tissues with PcAb-hNPC1 antibody. **(D)** Localization of BmNPC1 in BmE cells. BmE cells were permeabilized (a and b) and unpermeabilized (c and d). a and c was immunostaining with BmNPC1-C antibody, b and d was immunostaining with pre-immune rabbit serum as control. The late endosome was labeled by Rab7-mCherry (red, a2 and b2) and BmNPC1-C antibody (green, a1) in the permeabilized BmE cells. The plasma membrane was labeled by DiI (red, c2 and d2) and BmNPC1-C antibody (green, c1) in the unpermeabilized BmE cells. The nucleus was stained by DAPI. Bar, 5 μm.

To identify *B. mori* homologs of the *npc1* gene, the hNPC1 gene sequence (gi|255652944) was used to conduct a BLAST search in the *B. mori* genome database in NCBI. Results revealed that the gene (XP_012544312.1) encoding a protein of 1334 amino acids long shared the highest sequence similarity of 43% with hNPC1 (Supplementary Figure S1), indicating this gene is a potential homolog of a vertebrate NPC1 family member. This gene was referred as BmNPC1 in our study. Similar to hNPC1, structure prediction revealed that BmNPC1 protein contains 13 transmembrane-spanning helices and 3 large luminal loops, namingly domain A (residues 1 to 270), domain C (residues 408 to 645), and domain I (residues 881 to 1136) (Figure 1A); Like hNPC1, BmNPC1 protein includes three domains: the NTD domain (NPC1 NTD, residues 31 to 270), the SSD domain (Sterol-sensing domain, residues 674 to 828), and the patched domain (residues 1088 to 1289). Phylogenetically, BmNPC1 can be grouped in a single clade with *Apis cerana*, *Drosophila melanogaster*, and is relatively distant from NPC1s of *Saccharomyces cerevisiae* and *Toxoplasma gondii* (Supplementary Figure S1).

To further study the biological function of BmNPC1 and its role in baculovirus infection in insect cells, the full length (4 kb) coding sequence (CDS) of BmNPC1 was cloned from the *B. mori* cDNA library (Figure 1B); the sequence was 100% identical to the sequence of XP_012544312.1 published in the NCBI database. Furthermore, a rabbit polyclonal antibody against hNPC1 (PcAb-hNPC1) was used to determine protein expression and localization of BmNPC1. A single band of ∼140 kDa was detected in testis tissues by Western blot (Figure 1C). BmNPC1 proteins were localized in the plasma membrane and late endosomes in BmE cells as revealed by IFA (Figure 1D). Rab7-fused mCherry is a marker for late endosomes. Combined with previous studies that hNPC1 located on the late endosomes and lysosomes, suggest BmNPC1 also located on the late endosomes and lysosomes. Taken together, we demonstrate that the putative BmNPC1 gene (XP_012544312.1) shares high sequence and structural similarity with hNPC1 and was transcribed and translated in BmE cells, with the protein abundantly expressed in the plasma membrane and intracellular vesicles.

### The Potent NPC1 Antagonists, Imipramine and U18666A, Inhibited BmNPV Infection in BmE Cells

**FIGURE 2:**
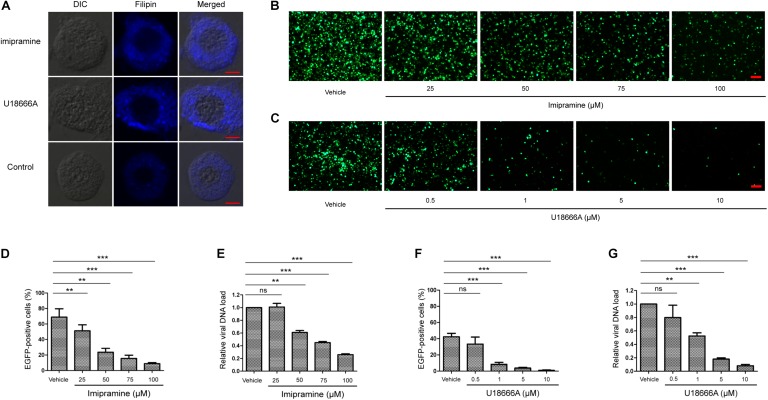
Cationic amphiphilic compounds imipramine or U18666A inhibits BmNPV replication in BmE cell. **(A)** The cholesterol staining of BmE cells with filipin. BmE cells were pretreated with imipramine (100 μM for 2 h) or U18666A (10 μM for 24 h) and stained by filipin (blue). Bar, 5 μm. BmE cells were pretreated with imipramine **(B)** or U18666A **(C)** for 2 and 24 h, respectively, followed by BmNPV-EGFP infection at a MOI of 1 for 72 h. Viral infection was indicated by positive EGFP expression in BmE cells. Bar, 200 μM. Quantification of BmNPV-EGFP infectivity in BmE cells pretreated with imipramine **(D)** or U18666A **(F)**. The percentage of EGFP positive cells was counted from at least five different fields. Fold decrease of viral DNA load in BmE cells pre-treated with imipramine **(E)** or U18666A **(G)** compared to control. The viral DNA load was analyzed by qPCR analysis using *gp41* primers, and the viral DNA load in cells treated with blank solvent was set as “1.” Relative copy numbers were calculated using *B. mori gapdh* as the internal control. ANOVA followed by Dunn’s Multiple Comparison Test were used for statistical analysis. ^∗^*P* < 0.05; ^∗∗^*P* < 0.01; ^∗∗∗^*P* < 0.001; n.s, non-significant, Means ± s.d. are shown (*n* = 3).

To investigate whether BmNPC1 is involved in BmNPV infection in insect cells, we first examined the effect of two small molecule NPC1 antagonists on BmNPV-EGFP infection in BmE cells. Both small molecules (imipramine and U18666A) have previously been shown to mimic the molecular phenotype of NPC disease and block the exit of cholesterol from late endosomal compartments (Côté et al., 2011; Wichit et al., 2017). When the BmE cells were treated with imipramine (100 μM for 2 h) or U18666A (10 μM for 24 h), filipin staining results showed that BmE cells presented cholesterol accumulation in the cytoplasm (Figure 2A), suggesting both inhibitors could block the intracellular cholesterol trafficking pathway. Then BmE cells were pretreated with imipramine (25–100 μM for 2 h) or U18666A (0.5 to 10 μM for 24 h) before the addition of BmNPV viral inoculum. The effect of drug pretreatment on viral infection was evaluated based both on the percentage of EGFP expression positive cells at 72 h post infection (p.i.), and the quantification of viral DNA accumulation in infected cells by qPCR. Imipramine and U18666A showed no evident cytotoxicity in BmE cells (Supplementary Figure S2), and drug-pretreatment resulted in a dose-dependent reduction in viral infectivity as demonstrated by the percentage of EGFP positive cells (Figures 2B,C). The reduction in EGFP expressing positive cells upon drug treatment was statistically significant at concentrations of 25–100 μM for imipramine and of 1–10 μM for U18666A (Figures 2D,F). Consistent with the quantification of EGFP positive cells, the viral load (represented by relative copy number of *gp41*) decreased significantly at concentrations of 50–100 μM for imipramine and at concentrations of 1–10 μM for U18666A. For BmE cells pretreated with 100 μM of imipramine or 10 μM of U18666A, the viral load was reduced to 27 and 10%, respectively, compared to the vehicle along treatment control (Figures 2E,G). In summary, we conclude that blockage of BmNPC1 function by small molecules can efficiently reduce BmNPV infection in BmE cells, indicating that functional BmNPC1 is required for BmNPV infection in insect cells.

### Knockdown of BmNPC1 Expression by RNAi Reduced Virus Infection in BmE Cells

**FIGURE 3:**
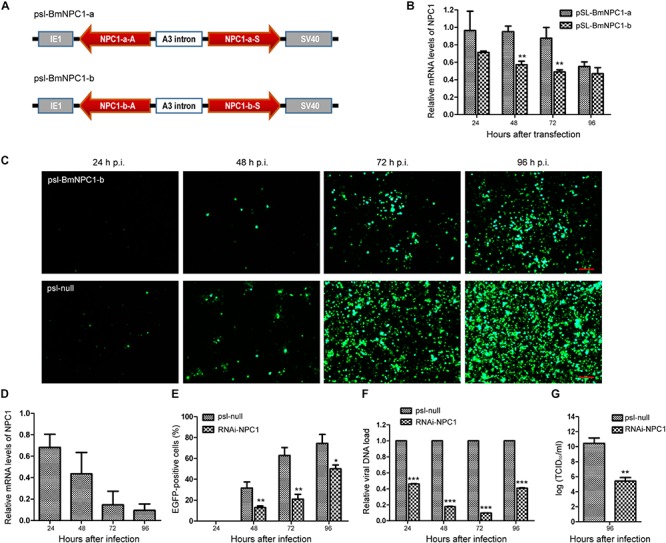
Silence of BmNPC1 inhibits BmNPV replication in BmE cell. **(A)** Schematic diagram of the BmNPC1 RNAi vector construction. IE1 indicates the *ie1* promoter of BmNPV. NPC1-a and NPC1-b are two NPC1 fragments, NPC1-a-S, NPC1-b-S, NPC1-a-A, and NPC1-b-A represents the sense fragment (S) and the antisense fragment (A) of NPC1-a and NPC1-b, respectively. These backbones were inserted into pSL1180 vector (“head to head”), the arrow direction of gene fragment represented 5′–3′. The A3 intron was a spacer cloned from the genomic DNA of *B. mori* Dazao. SV40 was the polyadenylation signal. **(B)** The effect of BmNPC1 RNAi transfection in BmE cell. BmE cell was transfected with psl-BmNPC1-a or psl-BmNPC1-b, and the relative mRNA levels of NPC1 was detected by qPCR at the indicated times. **(C)** Fluorescence microscopy of BmNPV infected BmE cells transfected with psl-BmNPC1-b or null RNAi at indicated times. Cells transfected with psl-BmNPC1-b or null RNAi were infected with BmNPV at a MOI of 3. BmNPV infection positive cells were indicated by EGFP expression. Bar, 200 μM. **(D)** Relative expression level of BmNPC1 in BmNPV-infected BmE cells pretreated with psl-BmNPC1-b compared to that of null RNAi vector. After transfection 48 h, pretreated BmE cells were challenged by BmNPV. qPCR was used to detect the expression of BmNPC1 in BmE cells transfected with psl-BmNPC1-b at indicated times. The relative expression level of each reaction was normalized to the endogenous control BmRPL3 (*Bombyx mori* ribosomal protein L3). **(E)** Quantification of BmNPV infection based on EGFP expression in BmE cells transfected with psl-BmNPC1-b and psl-null RNAi vector. The percentages of EGFP positive cells of five different fluorescent fields were counted in each group. **(F)** The relative viral DNA load in cells transfected with psl-BmNPC1-b and psl-null RNAi vector. The viral DNA load were determined by qPCR analysis using *gp41* DNA primers, the viral DNA loads in cells transfected with psl-null RNAi vector at the indicated times were set as “100%.” Relative copy numbers were calculated using *B. mori gapdh* as the internal control. **(G)** Virus titer determination by TCID50 end point dilution assays. Supernatants were harvested at 96 h p.i. from the cells infected with BmNPV for the virus titer titration. Virus infection was determined by positive EGFP expression in BmE cells by fluorescence microscopy. Independent-samples *T-*tests were used for statistical analysis. ^∗^*P* < 0.05; ^∗∗^*P* < 0.01; ^∗∗∗^*P* < 0.001; n.s, non-significant, Means ± s.d. are shown (*n* = 3).

To further validate whether BmNPV infection needs BmNPC1 expression, two sets of RNAi vectors targeting BmNPC1 (psl-BmNPC1-a and psl-BmNPC1-b) were constructed (Figure 3A), and independently transfected into BmE cells. The reduction of BmNPC1 expression at the mRNA level following BmNPC1-RNAi treatment was detected by qPCR at various time points post transfection (p.t.). psl-BmNPC1-b was selected to knock down BmNPC1 expression in BmE cells due to greater efficiency and duration in reducing the expression of BmNPC1 (40% of reduction compared to psl-null at 48 h, with the knock-down effect persisting until 96 h p.t.) (Figure 3B). BmE cells were treated with psl-BmNPC1-b or psl-null for 48 h prior to infection with BmNPV-EGFP. As shown in Figure 3C, viral infectivity as indicated by EGFP expression decreased significantly in psl-BmNPC1-b treated cells at 48, 72, and 96 h p.i. compared to that in control RNAi treated cells (Figure 3E). Furthermore, viral loads represented by relative copy number of *gp41* in BmNPC1 RNAi treated cells was less than 10% of that in control RNAi-treated cells at 72 h p.i. (Figure 3F). The viral titer (5.6 log_10_TCID_50_/ml) at 96 h p.i. as quantified by TCID_50_ in BmNPC1 RNAi treated cells was significantly lower compared to that in control cells (10.8 log_10_TCID_50_/ml, Figure 3G), the decrease of viral DNA load was due to the reduction of BmNPC1 expression (Figure 3D). Collectively, we conclude that knocking down BmNPC1 expression by RNAi in BmE cells can substantially reduce BmNPV infectivity.

### BmNPV Virus Binding and Entry in the Absence of BmNPC1

**FIGURE 4:**
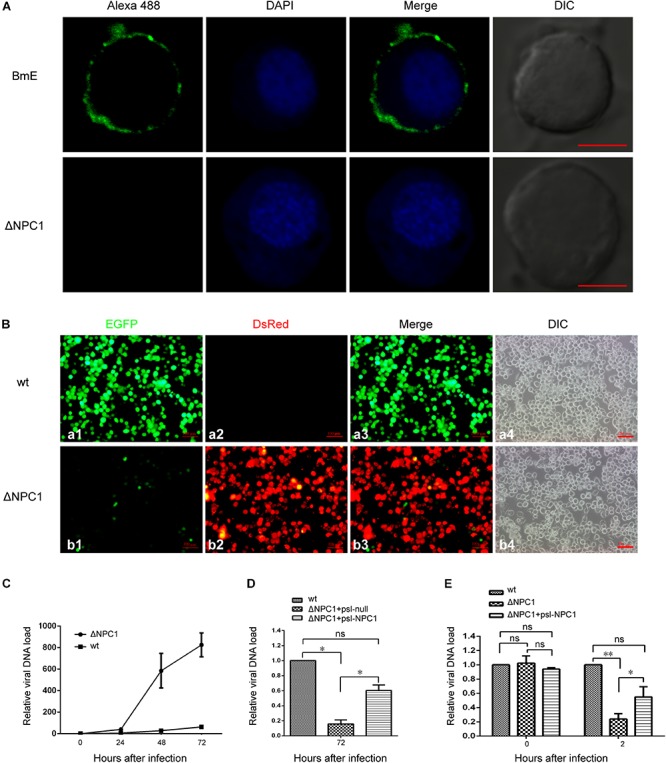
*Bombyx mori* Niemann-Pick C1 deficiency affect viral entry, but not viral adhesion. **(A)** Expression of BmNPC1 in wild type or BmNPC1-null BmE cells. The BmNPC1 was labeled by BmNPC1-C antibody (green fluorescence), and nucleus was stained by DAPI. The Bar, 5 μm. **(B)** Fluorescence microscopy of BmNPV infected wild-type and BmNPC1 knocked out BmE cells at 72 h p.i. BmNPV infection positive cells were indicated by EGFP expression and BmNPC1 knocked out BmE cells were indicated by DsRed expression. Bar, 100 μM. **(C–E)** Wild-type BmE cells and BmNPC1-null BmE cells (ΔNPC1) were inoculated with BmNPV, and cell samples were collected at indicated time. BmNPV viral loads were measured by q-PCR analysis using *gp41* DNA primers, and relative copy numbers were calculated using *B. mori gapdh* as the internal control. **(C)** The relative viral DNA load in NPC1-null cells and wild-type cells at indicated time. The viral DNA loads in wild-type cells at 0 h p.i. were set as “1.” **(D)** BmNPV-EGFP infection of ΔNPC1 BmE cell transfected by psl-NPC1. Wild-type, NPC1-null complemented with NPC1 or a vector control was infected with BmNPV-EGFP. The viral DNA loads in wild-type cells at the indicated times were set as “100%.” **(E)** The relative viral DNA load in NPC1-null cells and wild-type cells at 0 h p.i. and 2 h p.i. Pre-cooled NPC1-null cells, NPC1-null complemented with NPC1 cells and wild-type cells were inoculated with BmNPV-EGFP virus, and the relative viral DNA load were determined at 0 and 2 h post infection. The viral DNA loads in wild-type cells at the indicated times were set as “100%.” ANOVA followed by Dunn’s Multiple Comparison Test were used for statistical analysis. ^∗^*P* < 0.05; ^∗∗^*P* < 0.01; n.s., non-significant, Means ± s.d. are shown (*n* = 3).

To further determine which viral infection stages were influenced by BmNPC1 deficiency, we generated BmNPC1 null stable cell line by CRISPR-Cas9 genome engineering. Partial sequences of *Bmnpc1* gene was replaced by ie1-DsRed and A3-neo expression cassettes to abolish the function of BmNPC1 in BmE cells (Supplementary Figure S3B), BmNPC1 disrupted BmE cells expressed with red fluorescence were sorted by flow cytometry (Supplementary Figure S3C) and the mutation was confirmed by PCR amplification and sequencing (Supplementary Figure S3D). IFA results confirmed that NPC1 protein was knocked out in BmNPC1 null cells (Figure 4A). Then we tested the susceptibility of the NPC1-null cells to BmNPV-EGFP virus infection, and found that the percentage of EGFP positive cells among BmNPC1-null cells as indicated by the expression of red fluorescence decreased dramatically compared to the control cells at 72 h p.i. (Figure 4B). qPCR analysis showed that viral infectivity decreased significantly in NPC1-null cells at 48 and 72 h p.i. compared to that in control cells, indicating that virus infection was blunted in NPC1-null cells (Figure 4C). Transfection of NPC1 in NPC1-null BmE cells rescued virus loads, confirmed that the decreased virus load in NPC1-null cells was caused by NPC1 deletion (Figure 4D).

To further investigate at which step(s) viral infection is blocked when BmNPC1 expression is downregulated or knocked out, we examined virus binding and entry in the absence of BmNPC1 BmE cells. For this purpose, BmNPV-EGFP viruses at an MOI of 10 were incubated with pre-cooled NPC1-null cells, NPC1-null complemented with psl-NPC1 cells or wild-type cells for 1 h at 4°C followed by PBS washing to remove any unbound viruses, and cell surface bound viruses were then quantified by qPCR. As shown in Figure 4E, null group had a similar viral DNA load compared with that of control group, suggesting BmNPC1 deficiency could not affect on viral attachment. In previous work, the baculovirus capsids was found in cell nucleus at 2 h after infection (Ohkawa et al., 2010), then we examined virus entry at 2 h post internalization by shifting temperature to 28°C for 2 h after virus binding at 4°C for 1 h. We found that compare to control group, the virus load in NPC1-null cells was reduced to 20%, and NPC1-null cells could rescue virus load to 60% by complemented with psl-NPC1, indicating that viral entry as early as 2 h post internalization can be partially blocked when BmNPC1 is absent. Altogether, these data confirmed that BmNPC1 deficiency could markedly decrease viral entry possibly at a step post virus binding.

### BmNPV GP64 Protein Interacted With BmNPC1 Mainly via C Domain

**FIGURE 5:**
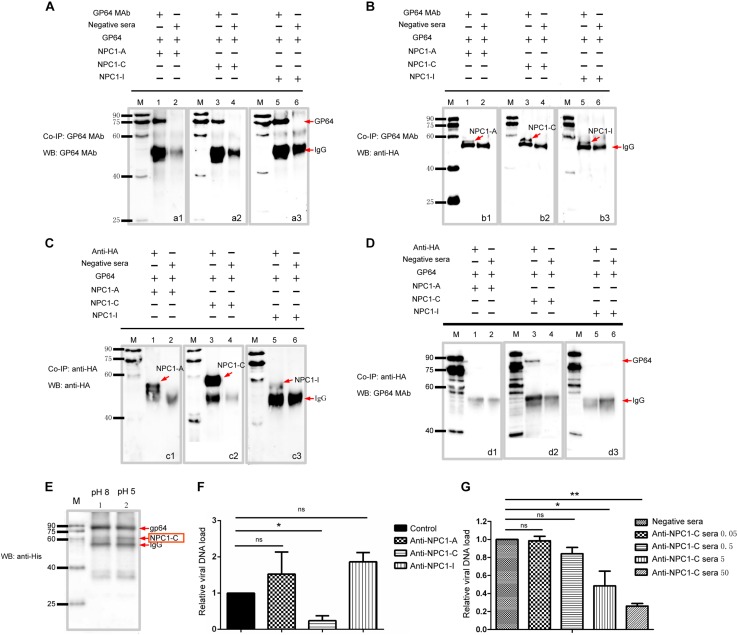
BmNPC1-C interacts with BmNPV GP64 and the antiBmNPC1-C antibody pretreatment reduces BmNPV infection. **(A,C)** The interaction between antibody and expressed proteins demonstrated by immunoprecipitation (IP). **(A)** Anti-GP64 monoclonal antibody was used for immunoprecipitation (IP) and immunoblot. A1, GP64 incubated with BmNPC1-A; A2, GP64 incubated with BmNPC1-C; A3, GP64 incubated with BmNPC1-I. **(C)** The anti-HA antibody was used for immunoprecipitation (IP) and immunoblot. C1, BmNPC1-A incubated with GP64; C2, BmNPC1-C incubated with GP64; C3, BmNPC1-I incubated with GP64. Lane M, EasySee Western marker. **(B,D)** BmNPC1 luminal loop domains interacted with BmNPV GP64 assay by CoIP. **(B)** Anti-HA monoclonal antibody was used for IP with BmNPC1-A, BmNPC1-C, or BmNPC1-I. B1, BmNPC1-A incubated with GP64; B2, BmNPC1-C incubated with GP64; B3, BmNPC1-I incubated with GP64. The anti-GP64 antibody was used for CoIP with BmNPC1-A, BmNPC1-C, or BmNPC1-I, and anti-HA antibody was used for immunoblot. **(D)** The anti-GP64 antibody was used for CoIP with GP64. D1, BmNPC1-A incubated with GP64; D2, BmNPC1-C incubated with GP64; D3, BmNPC1-I incubated with GP64. Lane M, EasySee Western marker. **(E)** The pH dependent interaction between GP64 and BmNPC1-C. Anti-GP64 antibody was used for IP with GP64, anti-His antibody was used for immunoblot. Red arrows indicated the corresponding proteins showed at the arrows back. Lane M, EasySee Western marker. Line 1, pH 8; Line 2, pH 5. **(F)** The relative viral DNA load in BmE cells preincubated with domain specific BmNPC1 antibodies. BmE cells preincubated with anti-NPCI-A, C, I domain specific antibody or control serum at a concentration of 10 μg/ml for 2 h before cells were infected with BmNPV-EGFP at a MOI of 1 for 2 h. The viral DNA loads were analyzed by qPCR using *gp41* DNA primers. Relative copy numbers were calculated using *B. mori gapdh* as the internal control. The viral DNA load in cells preincubated with negative serum was set as “1.” **(G)** The relative viral DNA load in BmE cells preincubated with concentration gradient BmNPC1 domain C antibodies. Concentration of BmNPC1-C antibody was 0.05, 0.5, 5, and 50 μg/ml, respectively. The viral DNA load in cells preincubated with negative serum was set as “1.” Relative copy numbers were calculated using *B. mori gapdh* as the internal control. ANOVA followed by Dunn’s Multiple Comparison Test were used for statistical analysis. ^∗^*P* < 0.05; ^∗∗^*P* < 0.01; n.s, non-significant, Means ± s.d. are shown (*n* = 3).

It has been shown previously that hNPC1 is able to interact with a primed form of EBOV glycoprotein GP, the primary glycoprotein responsible for receptor binding and fusion (Carette et al., 2011; Han et al., 2016). To investigate whether BmNPC1 contributes to BmNPV infection by interacting with glycoprotein GP64 during viral entry, we performed co-immunoprecipitation with *in vitro* expressed proteins (BmNPC1 domain A, C, I and BmNPV GP64). GP64-attached protein G agarose beads through mouse anti-GP64 antibody were incubated with BmNPC1 domain-specific proteins (domain A, C and I, respectively) for immunoprecipitation. The proteins that bound to the beads, i.e., the co-immunoprecipitation samples were then analyzed by Western blot. As shown in Figure 5A, GP64 proteins could be detected in the immune pellets by GP64 monoclonal antibody. The same immune pellets were subsequently probed with anti-HA antibody, and a specific band corresponding to the NPC1 domain A, C and I in each individual blot was shown in Figure 5B, and the band corresponding to NPC1-C displaying the strongest signal. Our results suggest that GP64 is able to interact with distinct domains (A, C, and I) of the NPC1 protein, with the strongest binding affinity for NPC1-C. Next, we performed the reciprocal co-immunoprecipitation experiments, in which NPC1 domain specific protein-attached beads were used to immune-precipitate GP64 proteins. The presence of NPC1-A, C or I in the retrieved beads was confirmed with anti-HA antibody (Figure 5C), however, the band corresponding to GP64 was only present in the NPC1-C-attached immune pellets (Figure 5D). To further confirm these interactions, we employed a yeast two-hybrid system (Y2H). Full-length GP64 was cloned into the pGBKT7 vector as bait, and BmNPC1-A, BmNPC1-C, BmNPC1-I were used as the prey. These Y2H screens revealed that BmNPC1-C interacted with full-length GP64, whereas BmNPC1-A or BmNPC1-I bound to GP64 with a weak affinity (Supplementary Figure S4). Collectively, these results demonstrate that domain C of BmNPC1 is sufficient for GP64 binding, while in comparison, the binding affinity of BmNPC1-A or BmNPC1-I to GP64 is relatively low.

Next, we investigated whether the interaction between BmNPV GP64 and BmNPC1 domain C was pH dependent. For this purpose, the Co-IP between BmNPV GP64 and BmNPC1-C was performed at pH 5 or 8. NPC1-C and GP64 proteins were expressed with His tag *in vitro*, and then the protein mixtures were incubated with protein G beads that had been conjugated with mouse monoclonal antibody to GP64. Anti-His antibody was used to detect both GP64 and NPC1-C at the same blot for more accurate comparison. As shown in Figure 5E, we found a stronger BmNPC1-C signal at pH 5 compared to that at pH 8, indicating that BmNPV GP64 binding affinity to BmNPC1-C was enhanced at a lower pH environment.

To further demonstrate that viral entry mainly relies on C domain of BmNPC1, we assessed the susceptibility of BmNPV infection in the presence of BmNPC1 domain-specific antibodies. Specific antibodies targeting extracellular loops A, C, and I of *Bombyx mori* Niemann-Pick C1 were incubated with BmE cells at 28°C for 2 h prior to virus infection, cells treated with a negative IgG were used as control. Intriguingly, compared to the control group, BmNPV load was significantly reduced in BmE cells treated with antibody specific for BmNPC1-C (Figure 5F), and treatment of cells with BmNPC1-C sera decreased the amount of the BmNPV-EGFP in BmE cells in a dose-dependent manner (Figure 5G), but the viral DNA load was no significantly changed in BmE cells treated with antibodies BmNPC1-A or BmNPC1-I sera (Figure 5F). Together, these data suggest that NPC1 domain C, but not domain A and I, is essential for GP64 mediated BmNPC1 infecton in BmE cells.

## Discussion

Baculoviruses have been widely used as important biological agents for controlling insect populations, and powerful biological tools for gene delivery and expression; a better understanding of the molecular mechanisms and host factors involved in baculovirus virus entry is of great significance in bioscience and biotechnology. In this study, we used the well-studied baculovirus BmNPV as a tool to investigate the host factor requirements for baculovirus infection, and found that BmNPC1, the hNPC1 homolog in insect cells, is indispensable for BmNPV infection in insect cells. Our results, together with previous work identifying a role for hNPC1 as an intracellular receptor for EBOV entry, have revealed that the conserved host protein NPC1, essential for cholesterol homeostasis, has been exploited by a group of divergent viruses for entry.

As an essential component of cellular membranes, cholesterol is one of the most important lipids for maintaining cell viability, cell signaling and physiology (Incardona and Eaton, 2000; Goldstein et al., 2006). Two major proteins that function in intracellular cholesterol transport are NPC1 and NPC2. NPC2 is the central shuttle in a unidirectional transfer pathway that mobilizes cholesterol to NPC1, leading to NPC1 export of cholesterol from late endosomes (Infante et al., 2008; Kwon et al., 2009; Deffieu and Pfeffer, 2011; Gong et al., 2016). Recently, it has been demonstrated that filoviruses, including Ebola and Marburg viruses, utilize NPC1 as intracellular receptors for entry (Carette et al., 2011). Interestingly, in addition to EBOV (family: *Filoviridae*), hepatitis C virus (family: *Flaviviridae*) entry also requires the cholesterol trafficking receptor Niemann-Pick C1-Like 1 (NPC1L1) (Sainz et al., 2012), a NPC1 paralog which serves in cellular cholesterol absorption and homeostasis on the apical surface of intestinal enterocytes and human hepatocytes (Altmann et al., 2004; Yu, 2008). Moreover, the release of HIV-1 (family: *Retroviridae*) (Tang et al., 2009) and Chikungunya virus (family: *Togaviridae*) (Wichit et al., 2017) is impaired in cells from patients with NPC disease. U18666A or imipramine, which mimics a NPC-deficient phenotype, also strongly inhibits the replication of several *flaviviridae* family members, including Zika, West Nile, and Dengue viruses (Jupatanakul et al., 2014; Wichit et al., 2017). In our study, we present evidence that baculovirus utilizes the NPC1 homolog to enter into insect cells. Although the enveloped viruses described above belong to distinct viral families (*Baculoviridae*, *Filoviridae*, *Togaviridae*, and *Flaviviridae)*, and possess different types (dsDNA, +ssRNA or −ssRNA) and sizes of viral genomes, they invariably hijack NPC1 in the cholesterol transport pathway to initiate infection. We speculate that NPC1 is the common entry host factor for many enveloped viruses during cellular infection. However, it should be noted that infection with some enveloped viruses from *Rhabdoviridae* [vesicular stomatitis virus (Carette et al., 2011)] or *Orthomyxoviridae* [infiuenza A virus (Aminibavilolyaee et al., 2013)] occurs independently of NPC1.

However, despite both EBOV and baculovirus requiring NPC1 for viral entry, NPC1-dependent baculovirus infection of insect cells exhibits unique features. The EBOV glycoprotein GP is a Class I fusion protein (Vigant et al., 2015). During virus entry, EBOV is internalized into host cells by receptor binding-mediated macropinocytosis and is then transported to late endosomes (Nanbo et al., 2010; Mulherkar et al., 2011). In the endosome, cathepsin B-mediated cleavage removes mucin and glycan cap domains from the GP protein and exposes a hydrophobic cavity, which can mediate binding to the hNPC1 C domain (Miller et al., 2012; Gong et al., 2016; Han et al., 2016). The specific binding between hNPC1 and GPcl was hypothesized to facilitate fusion between vial membrane and host endosomal membranes (Gong et al., 2016). In contrast, the BmNPV glycoprotein GP64 is a Class III fusion protein, and viral fusion is triggered by an acidic pH without proteolytic cleavage (Kadlec et al., 2008). We show here that BmNPC1 proteins are located at both the cell plasma membrane and intracellular compartments. BmNPC1 is capable of binding to GP64 at a neutral pH suggesting that BmNPC1 may serve as a host factor to facilitate virus attachment to the cell surface. However, we found that BmNPC1 deficiency could not reduce the viral binding. Then we demonstrate that BmNPC1 deficiency could significantly decrease viral entry at 2 h p.i, suggesting that absent of BmNPC1 in the late endosome and lysosome influenced virus escaped from the lysosome through viral fusion. The binding between BmNPC1 and GP64 was enhanced at a lower pH suggesting that low pH triggered-conformational changes in the GP64 protein may expose certain epitopes or domains to allow better accessibility by BmNPC1, and the specific binding between GP64 and BmNPC1 may ultimately enable GP64 to maintain a certain structure which is essential for targeting its fusion domain into the endosomal membranes. However, the detailed mechanism of BmNPC1 protein in baculovirus entry will rely on future crystallographic examination of the BmNPC1 and GP64 complex. Such studies will ultimately shed light on how a wide range of enveloped viruses utilize the shared host factor NPC1 as part of fusion triggers for intracellular entry.

Baculovirus can transduce a broad range of cells including both vertebrate and invertebrate cells, indicating that conserved host factors may be involved in viral entry. In our study, we have only examined the role of NPC1 in viral entry in insect cells; it will be of great interest in the future to assess whether NPC1 is involved in viral entry in mammalian cells. Furthermore, to identify the role(s) of BmNPC1 in various steps of viral entry is warranted. In summary, our study demonstrated that BmNPV entry requires the cholesterol transporter BmNPC1 as the host factor for baculovirus infection. Firstly, baculovirus binds to the cell surface with some unknown attachment factors, and is then internalized and trafficked to late endosomes. In the acidic pH environment of endosomes, GP64 undergoes conformational changes to expose certain domain(s) for better access by BmNPC1. The specific binding between GP64 and BmNPC1 subsequently facilitates viral fusion events to allow nucleocapsids to be released into the cytoplasm. Our work fills an important gap in baculovirus research, and identifies a new antiviral target against baculovirus infection. Elucidation of baculovirus entry mechanisms will further facilitate the application of baculovirus systems in eukaryotic gene delivery. As the cholesterol transporter NPC1 is shared by several viral families, this work provides a new avenue of inquiry that NPC1 may represent a common entry factor for many enveloped viruses entry.

## Author’s Note

This manuscript has been released as a Pre-Print at bioRxiv (Li et al., 2018).

## Data Availability Statement

All datasets generated for this study are included in the article/Supplementary Material.

## Ethics Statement

The animal study was reviewed and approved by the Laboratory Animals Ethics Review Committee of Southwest University.

## Author Contributions

ZL conceived and designed the experiments, and drafted the manuscript. YF, XM, and YZ performed the experiments. JB, TL, ML, and JC contributed the reagents, materials, and analysis tools. JW and QH analyzed the data and organized the original figures. GP, CL, and ZZ conceived the study and critically revised the manuscript. All authors read and approved the final version of the manuscript.

## Conflict of Interest

The authors declare that the research was conducted in the absence of any commercial or financial relationships that could be construed as a potential conflict of interest.

## Footnotes

1 http://www.cbs.dtu.dk/services/TMHMM-2.0/

2 http://smart.embl-heidelberg.de/

3 http://sirna.wi.mit.edu/home.php

4 http://crispr.dbcls.jp/
