# Supplementary material for: Baculovirus Utilizes Cholesterol Transporter NIEMANN–Pick C1 for Host Cell Entry
Source: Front Microbiol. 2019 Dec 5;10:2825. doi: 10.3389/fmicb.2019.02825 (PMC6906155; doi:10.3389/fmicb.2019.02825)
Supplement: Supplementary file 1 [file Data_Sheet_1.pdf]

Supplementary Material

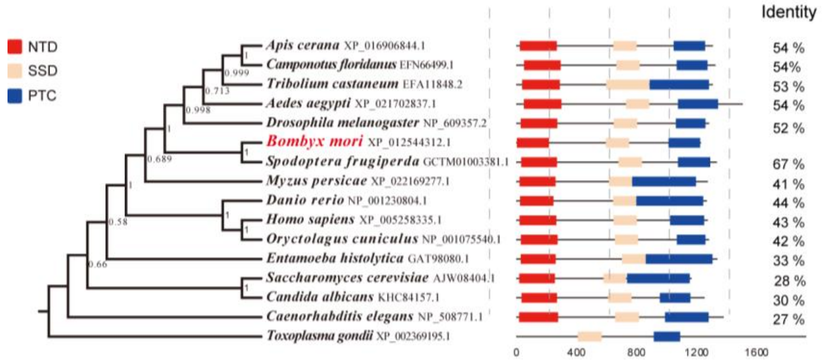

Fig S1 | Phylogenetic tree of BmNPC1. Phylogenetic tree was constructed using neighbor-joining (NJ) analysis with MEGA version5. The NPC1 proteins among different species were listed on the left side. GenBank accession number for each sequence is shown in the parenthesis. Numbers on horizontal lines in the trees indicate the confidence percentages of the tree topology from bootstrap analysis of 1000 replicates. Schemes show the main structural characteristics of NPC1 from distinct organisms (the right side); following is the corresponding identity compare to the BmNPC1 sequence. Red rectangle: NTD; pink rectangle: SSD; blue rectangle: PTC.

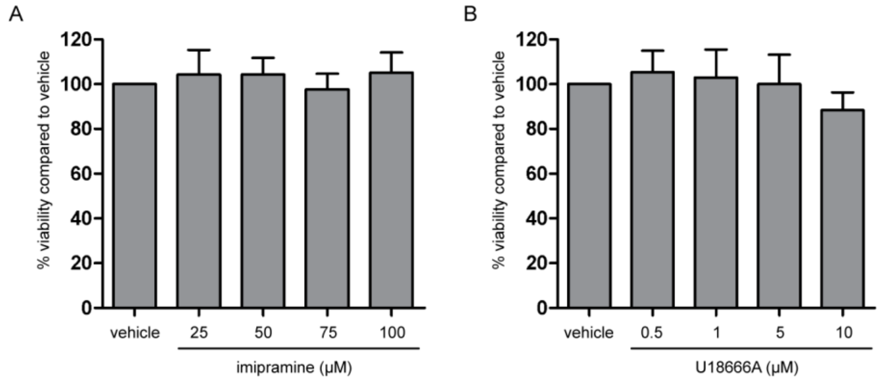

Fig S2 | The effect of NPC1 antagonists on BmE cells viability. BmE cells were treated with methyl alcohol (vehicle), 25, 50, 75 and 100 μM of imipramine **(A)** or culture (vehicle), 0.5, 1, 5 and 10 μM of U18666a **(B)**. After 2 h (A) or 24 h (B), cell viability was determined by CCK-8 assay. BmE cells treated with blank solvent were set as “100%”. Means ± s.d. are shown (n=3).

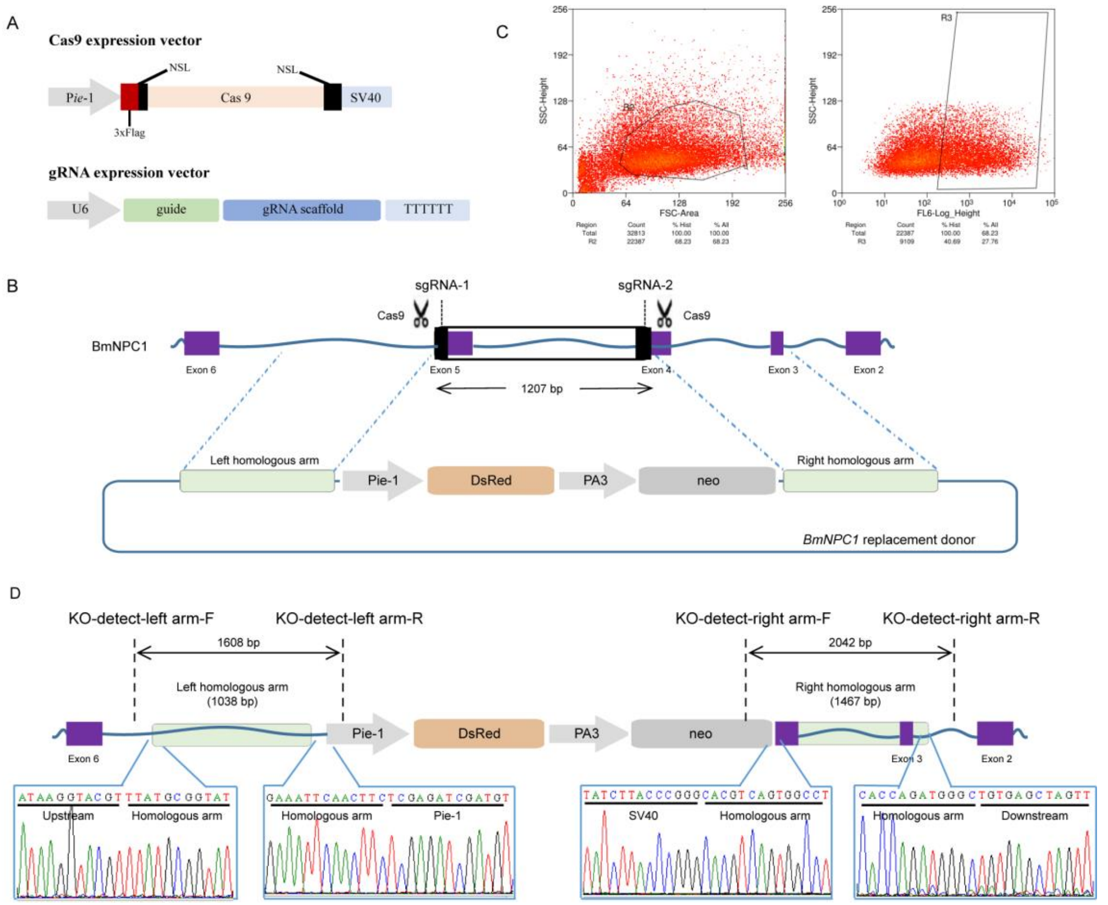

Fig S3 | Generation of BmNPC1 knockout BmE cell using CRISPR/CAS9 system. **(A)** Schematic structure of Cas9 and gRNA expression vector for BmE cell. Cas9 was driven with *ie-1* promoter (gray) and ended with SV40 (wathet) terminator. Chimeric gRNA contained U6 promoter (gray), chimeric site (green), gRNA1 scaffold (blue), and terminal sequence (TTTTTT). **(B)** Schematic for homologous recombination (HR) mediated by Cas9. Homologous arms (light green) were PCR amplified from BmE cells genomic DNA with specific primers (Supplementary Table S1). The Left-HR, DsRed expression cassettes, *neo* expression cassettes and Right-HR were sequentially cloned into the pBluescript II KS (-) vector to generate donor plasmid. BmE cells genomic DNA was cut by Cas9, and about 1207 bp of BmNPC1 was replaced through homologous recombination (HR) to completely abolished expression of BmNPC1. **(C)** BmNPC1 disrupted BmE cells labeled with red fluorescence were sorted by flow cytometry. **(D)** Verification of BmNPC1 knocked out BmE cells by PCR and sequencing. The sequences of PCR amplification products with specific primers (Supplementary Table S1) from the BmNPC1 disrupted BmE genomic DNA indicated the partial removal of *BmNPC1* and replaced by DsRed and *neo* expression cassette.

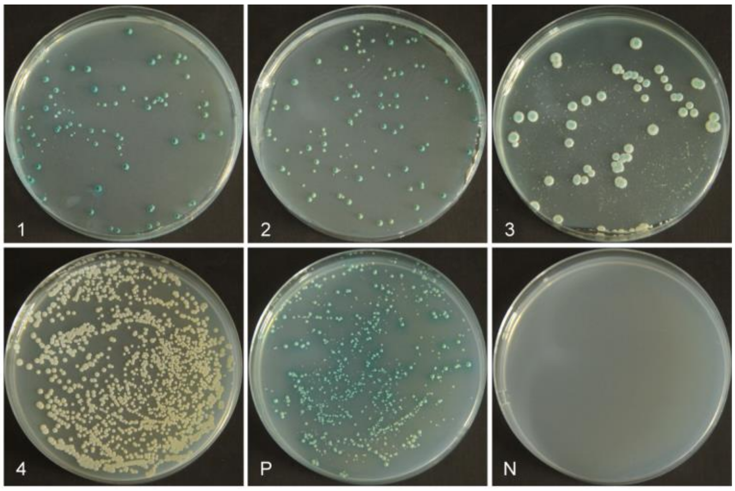

Sample	Plasmids	Interaction
1	pGADT7-BmNPC1-A/pGBKT7-GP64	Yes
2	pGADT7-BmNPC1-C/pGBKT7-GP64	Yes
3	pGADT7-GP64/pGBKT7-BmNPC1-C	Yes
4	pGADT7-BmNPC1-I/pGBKT7-GP64	No
P	Positive control pGBKT7-53/pGADT7-T	Yes
N	Negative control pGBKT7-lam/pGADT7-T	No

Fig S4 | Yeast two-hybrid assay for determination the interaction of BmNPC1-A, BmNPC1-C and BmNPC1-I with GP64. pGADT7-BmNPC1-A (prey)/pGBKT7-GP64 (bait), pGADT7-BmNPC1-C (prey)/pGBKT7-GP64 (bait), pGADT7-BmNPC1-I (prey)/pGBKT7-GP64 (bait), pGADT7-GP64 (prey)/pGBKT7-BmNPC1-C (bait) constructs were transformed into competent yeast cells, respectively. A number of independent blue colonies including the pGADT7-BmNPC1-A (prey)/pGBKT7-GP64 (bait) (sample 1), pGADT7-BmNPC1-C (prey)/pGBKT7-GP64 (bait) (sample 2) and pGADT7-GP64 (prey)/pGBKT7-BmNPC1-C (bait) (sample 3) constructs grew on SD plates lacking Leu, Trp, His, and Ade and containing X-α-Gal. However, the colony pGADT7-BmNPC1-I (prey)/pGBKT7-GP64 (bait) could grow on the above SD plate but did not turn blue. This demonstrates the interactions of BmNPC1-A and BmNPC1-C with GP64, while BmNPC1-I did not interact with GP64. Positive-control pGBKT7-53/pGADT7-T (P) and negative-control pGBKT7-lam/pGADT7-T (N) reactions are provided for each group.
